# Foodborne Illness Incidence Rates and Food Safety Risks for Populations of Low Socioeconomic Status and Minority Race/Ethnicity: A Review of the Literature

**DOI:** 10.3390/ijerph10083634

**Published:** 2013-08-15

**Authors:** Jennifer J. Quinlan

**Affiliations:** Department of Nutrition Sciences, Drexel University, 1505 Race St., Mail Stop 1030, Philadelphia, PA 19102, USA; E-Mail: jjq26@drexel.edu; Tel.: +1-215-762-8456; Fax: +1-215-762-4080

**Keywords:** foodborne illness, food safety, low income, minority, vulnerable populations, food desert, ethnic foodservice, socioeconomic, food desert

## Abstract

While foodborne illness is not traditionally tracked by race, ethnicity or income, analyses of reported cases have found increased rates of some foodborne illnesses among minority racial/ethnic populations. In some cases (*Listeria*, *Yersinia*) increased rates are due to unique food consumption patterns, in other cases (*Salmonella*, *Shigella*, *Campylobacter*) it is unclear why this health disparity exists. Research on safe food handling knowledge and behaviors among low income and minority consumers suggest that there may be a need to target safe food handling messages to these vulnerable populations. Another possibility is that these populations are receiving food that is less safe at the level of the retail outlet or foodservice facility. Research examining the quality and safety of food available at small markets in the food desert environment indicates that small corner markets face unique challenges which may affect the quality and potential safety of perishable food. Finally, a growing body of research has found that independent ethnic foodservice facilities may present increased risks for foodborne illness. This review of the literature will examine the current state of what is known about foodborne illness among, and food safety risks for, minority and low socioeconomic populations, with an emphasis on the United States and Europe.

## 1. Introduction

Foodborne illness continues to be a public health burden, with most recent estimates of 9.4 million cases per year in the United States, resulting in 1,351 deaths [1]. Incidence rates of foodborne illness have not traditionally been tracked by race, ethnicity or income. A limited number of studies have found that low income populations are more likely to experience greater rates of gastrointestinal illness [2,3,4,5,6]. There is also growing evidence that individuals of minority racial and ethnic groups suffer from greater rates of some foodborne illnesses [7,8,9,10]. The Foodborne Diseases Active Surveillance Network (FoodNet) [11], quantifies and monitors the incidence of laboratory-confirmed cases of *Salmonella*, *Campylobacter*, *Listeria*, Shiga-toxin producing *E. coli*, *Shigella*, *Yersinia* and *Vibrio*. The FoodNet catchment area was not chosen to equally represent all racial and ethnic groups and even in the expanded FoodNet population, Hispanics and those living below the poverty level are underrepresented when compared to the general American population (6% *vs*. 12%, and 11 *vs*. 14%, respectively) [12]. Over the past decade, analysis of FoodNet tracking data to examine the burden of foodborne illness on minority racial and ethnic populations has revealed trends related to their demographics. Additionally, since 2008 FoodNet final reports each year have reported incidence rates of bacterial pathogens by race and ethnicity [13].

If, as emerging data suggest, low income and minority populations experience greater rates of foodborne illness, the question arises as to where in the farm-to-fork continuum these populations might be experiencing greater risk of exposure to foodborne pathogens. In some cases, epidemiological evidence has made it clear that a particular food consumption pattern among a population resulted in increased exposure to a particular pathogen. This was the case for *Yersinia* and African Americans (chitterling consumption) as well as *Listeria* and Hispanic populations (fresh Mexican style cheese consumption) [14,15,16]. Increased incidence of *Salmonella*, *Shigella* and *Campylobacter* infections however, do not appear to be attributable to a single food source; thus, a logical first place to look is with the consumers themselves and their knowledge and behaviors related to food safety. A growing body of research has focused on the handling of foods by consumers of minority and low income populations [17,18,19,20,21,22,23]. This research has identified some safe food handling knowledge and behavior gaps which are consistent with those seen in the general population as well as some which are unique to these populations. 

Another possibility of where minority populations may experience greater risks for foodborne illness is at the food retail or food service level. A growing body of public health research [24,25,26] has demonstrated that low income and minority populations have different patterns of access to food at the retail level. This concept has been recognized and defined by the U.S. Department of Agriculture as “Food Deserts” where there is a lack of large supermarkets and tends to be an abundance of smaller grocers, convenience and fast food retailers [27,28]. A small body of research has begun to attempt to assess the food safety risks of food deserts and the small independent retailers they are made up of through a combination of survey at the retail level as well as use of inspection violation rates as a proxy for safety [29,30,31,32,33]. 

This review will summarize what is known about differential rates of foodborne illness as well as food handling and food access by minority consumers in an effort to identify needs for further research in this emerging area. This review is limited to literature in the English language. Emphasis is on the United States and Europe where published data indicate differential rates of foodborne illness among different populations. Research regarding consumer food handling and retail food access in countries other than the U.S. and Europe is not included in this review so that sections relating to incidence rates of illness, food access, and food handling all reflect roughly the same populations. Additionally, this review is limited to microbial foodborne illness and other non-infectious food safety risks such as chemicals, allergens, *etc*. are not covered. Research gaps and strengths may serve to identify best ways for researchers in other countries to proceed in examining these issues in their own cultures and among their unique minority and low income populations. 

## 2. Incidence Rates of Foodborne Illness among Low Socioeconomic Status and Racial and Ethnic Minority Populations

Definitions of race and ethnicity will vary for different countries. For the purpose of this review the races and ethnic groups discussed are those tracked by FoodNet where differences in incidence of foodborne illness have been reported. These include Caucasian, African American and Asian. The only ethnicity tracked by FoodNet is Hispanic. An individual who identifies as Hispanic ethnicity may identify as any race. While FoodNet data does not track incidence of foodborne illness by income, both African American race and Hispanic ethnicity have significantly greater percent of their populations in poverty (25.8 and 23.2%, respectively) than either Caucasians or the general population (11.6 and 14.3%, respectively). Poverty in the Asian population is 11.7%, close to that seen in the Caucasian population and below the general population [34]. 

Overall, FoodNet data as well as data from the U.S. National Notifiable Diseases Surveillance System [10] indicate that rates of illness among minority populations as compared to Caucasians vary by pathogen. That is, minority populations appear to suffer from greater rates of some pathogens (*Salmonella*, *Shigella*, *Listeria monocytogenes* and *Yersinia enterocolitica*), but appear to be less likely than Caucasians to experience illnesses due to *E. coli* O157:H7. Epidemiology of *Campylobacter* continues to remain unclear [35] but rates of campylobacteriosis remain lowest in African Americans when compared to all other demographics. Finally, many infections commonly transmitted through food (*i.e.*, norovirus) are not monitored by FoodNet because they are not routinely identified in clinical laboratories [36]. Therefore the data presented in this review will focus on the bacterial pathogens mentioned above, where differences in rates among different populations have been identified. 

### 2.1. Salmonella

FoodNet data from 1998 to 2000 indicated that the incidence of *Salmonella enteric* serovar *Enteritidis* infection was highest among African Americans (2.0/100,000 population), followed by Hispanics (1.2/100,000 population) and then Caucasians (1.2/100,000 population) [9]. Analysis of 2000 FoodNet data indicated that the incidence of *Salmonella enteric* serovar *typhi* was greater in Hispanics and Asians than Caucasians [8]. FoodNet data from 2008 to 2011 generally support the trend that minority populations suffer from a greater incidence of salmonellosis than Caucasians (Table 1). Chang *et al*. [10] performed an ecological analysis of sociodemographic factors associated with the three most commonly reported nationally notifiable enteric bacterial diseases in the U.S., salmonellosis, shigellosis and *E. coli* O157:H7. Data from the U.S. National Notifiable Diseases Surveillance System for infections reported in all U.S. counties from 1993 to 2002 was analyzed. Consistent with FoodNet data, it was found that percent African American and Hispanic population were positively associated with incidence of salmonellosis as was percent urban and number of food handlers in the population. A study of Italian children also previously associated “low social class” with incidence of *Salmonella* infection [3].

**Table 1:** Incidence rates (per 100,000 population) of bacterial causes of foodborne illness by race and ethnicity from 2008–2011 (data obtained from FoodNet Final Reports 2008–2011 [13]).

	Year	Caucasian	African American	Hispanic	Asian/Pacific
*Campylobacter*	2008	8.9	2.00	10.73	8.94
	2009	10.13	3.31	10.21	9.27
	2010	11.40	2.99	11.46	8.54
	2011	12.18	3.64	10.86	9.80
				
*Salmonella*	2008	11.1	7.4	15.5	11.07
	2009	12.05	13.39	12.93	14.94
	2010	14.64	16.19	14.61	15.62
	2011	13.85	14.02	13.4	15.82
				
*Shigella*	2008	3.73	6.67	8.64	1.75
	2009	2.26	6.95	5.65	1.95
	2010	2.02	7.95	5.25	1.67
	2011	1.96	6.22	4.34	1.14
				
*Listeria*	2008	0.19	0.19	0.53	0.33
	2009	0.33	0.26	0.44	0.14
	2010	0.27	0.19	0.30	0.37
	2011	0.29	0.25	0.39	0.37
				
*E. coli*	2008	1.05	0.26	0.67	0.71
	2009	1.04	0.37	0.71	0.64
	2010	1.03	0.39	0.60	0.54
	2011	1.05	0.38	0.70	0.41
				
*Yersinia*	2008	0.22	0.25	0.36	0.47
	2009	0.21	0.37	0.28	0.73
	2010	0.22	0.60	0.23	0.37
	2011	0.30	0.36	0.20	0.53

### 2.2. Campylobacter

The epidemiology of *Campylobacter* infections was explored utilizing FoodNet data from 1996 to 1999 and the average incidence over all four years was found to be greatest among Hispanic and Asian populations (31.6 and 33.5/100,000 population, respectively), while Caucasian populations had an incidence of 21.9/100,000 population and African Americans had the lowest incidence of 13.0/100,000 population [37]. While overall rates of *Campylobacter* have declined since FoodNet data began being recorded [13,37], the epidemiology of *Campylobacter* remains unclear, with unexplainable geographic variation [35]. One consistency however is that incidence rates of campylobacteriosis among African Americans remain low when compared to all other demographics over the past four years reported by FoodNet (Table 1) at 2.00–3.64/100,000 population. Over the same four year time period incidence rates (per 100,000 persons) among Caucasians have ranged from 8.91 to 12.18, among Hispanics have ranged from 10.21 to 11.46, and among Asians have ranged from 8.54 to 9.8. These data indicate that while African Americans have the lowest incidence of *Campylobacter*, Hispanics tend to have the highest incidence. It is unclear why African Americans appear to consistently demonstrate such low incidence of campylobacteriosis. One possibility is that this population may have greater immunity to *Campylobacter*. Immunity to *Campylobacter* has been established both in young children in developing countries [38,39,40] as well as with the consumption of raw milk [41]. It is not clear from FoodNet data whether African American children have greater rates of campylobacteriosis than children of other races and ethnicities, which may result in immunity among the African American population. Alternatively, the possibility exists that exposure to *Campylobacter* is through sources other than food such as direct contact or the environment. Recent studies have identified environmental factors associated with increased risks for campylobacteriosis in addition to the known risks of certain food consumption and exposure to farm animals [42,43,44]. 

### 2.3. Shigella

Analysis of FoodNet data from 1996 to 1998 found that the incidence of *Shigella* infection was greater in African Americans and Hispanics (4.1 and 11.2/100,000 population, respectively) when compared to Caucasians (1.6/100,000 population) [7]. Similarly, surveillance of a selected catchment area of the FoodNet states and counties in 2000 found that the incidence of *Shigella* infection was greater in Hispanics and African Americans but not Asians when compared to Caucasians [8]. Recent FoodNet data (Table 1) continues to show that Hispanics and African Americans, but not Asians, experience greater incidence of *Shigella* when compared to Caucasians. Chang *et al*. [10] found that percent African American, percent Hispanic, percent urban population and number of food handlers in the population were all positively associated with incidence of shigellosis [10]. It should be noted that the proportion of illnesses transmitted by non-food routes differs by pathogen and *Shigella* may have a high rate of fecal-oral transmission [36]. 

### 2.4. Listeria

*Listeria* has generally been found to be attributable to consumption of a number of high risk foods including sliced deli meats, raw milk, raw milk cheeses and unheated frankfurters [45]. High incidence of listeriosis among pregnant Hispanic women is an example of where a food culture contributes to increased rates of a foodborne illness. Consumption of fresh Mexican-style cheeses has been associated with increased risks of listeriosis among both pregnant and non-pregnant Hispanics [15,46,47,48]. Analysis of incidence rates of *Listeria* infection identified by FoodNet found that the overall annual incidence of listeriosis ranged from 0.25 to 0.32 cases per 100,000 populations between the time periods of 2004 to 2006 and 2007 to 2009. Over the same time periods incidence of pregnancy-associated listeriosis increased from 5.09 to 12.37/100,000 for Hispanic women and 1.74 to 2.80/100,000 for non-Hispanic women [49]. Therefore despite education efforts targeted toward these populations [50,51] there continues to be a need to educate both Hispanic and non-Hispanic pregnant women of the risks of *Listeria*. It should be noted that other significant risk factors for listeriosis include immunocompromised status as well as increasing age [45,49,52]. A recent analysis of FoodNet data found that within the 60–69 year old age group the Relative Risks (RR) for Hispanics was lower (6.3) than the RR for non-Hispanics (17.6) in the same age group [52]. Incidence of listeriosis in England between 2001–2007 was found to be highest among the most deprived areas when compared to the most affluent [6].

### 2.5. E. coli O157:H7

Chang *et al*. [10] found that while *E. coli* O157:H7 infections were not associated with percent African American, Hispanic or urban population, number of food handlers in the population was positively associated with *E. coli* O157:H7 infection. *E. coli* O157:H7 infection was also positively associated with percent population male, percent population living on a farm as well Midwest region and West region. Recent FoodNet data consistently show Caucasians suffering from the greatest incidence rates of *E. coli* O157:H7 infection and African Americans the lowest (Table 1). This is consistent with multiple studies that have found Caucasians report eating raw foods, including raw beef, more often than minority consumers [53,54]. Examination of the epidemiology of verotoxigenic *E. coli* in Finland found that in addition to proportion of fresh water per area and proportion of cultivated land per area, the proportion of low income households with children was also associated with an increased incidence of verotoxigenic *E. coli* infections [5].

### 2.6. Yersinia enterocolitica

While the overall incidence of *Yersinia* infections is fairly low when compared to that of other pathogens, historically it was the highest among African American infants and children [14]. Similar to *Listeria*, this is another example of where a food culture has resulted in an increased rate of foodborne illness in a population. The high incidence of yersiniosis among African American infants has been linked to the seasonal production of chitterlings (boiled large intestines of pigs following removal of fat and fecal material) among African American families, particularly in the South [55,56,57]. Following education interventions to advise proper handling and preparation of chitterlings (*i.e.*, www.health.state.ga.us/archives/pdfs/Chitlins_flyer.pdf) it has been reported that the incidence rate among African American children declined from 41.5/100,000 population in 1996 to 3.5/100,000 population in 2009 [55]. Recent FoodNet data (Table 1) indicates that there is still a slightly higher incidence among African Americans and Asians when compared to Caucasians. For the cases where race was reported between 1996 to 2000, the average age of Caucasians (49% of cases) was 35 years, while the average age of African Americans (40% of cases) was seven months and the average age of Asian/Pacific Islanders (10% of cases) was 3 years [55].

When comparing incidence rates of any disease between populations of different demographics it is important to understand that health care seeking behaviors vary between populations of different race, ethnicity and income levels. That is, some populations may be more or less likely to seek medical attention and therefore obtain confirmation of a foodborne infection. With respect to healthcare seeking behavior and stool sample submission in the FoodNet population it was found that both having health insurance as well as income below $25,000/year were associated with healthcare seeking behavior. Submission of a stool sample was more likely if an individual had either bloody diarrhea or diarrhea for greater than three days. It was concluded therefore that laboratory based surveillance may over-represent those with bloody diarrhea and longer diarrhea duration [58].

Overall the data support the concept that minority and low SES populations experience greater rates of many foodborne illnesses. In the cases of *Listeria* and *Yersinia*, education efforts related to cultural food preferences have reduced, but not eliminated the disparity in rates of the these infections in Hispanics and African Americans, respectively. This would indicate a need to continue and possibly increase education efforts for these populations. The higher rates of *Salmonella*, *Shigella* and *Campylobacter* in minority populations do not appear to be related to a single food source or consumption pattern and therefore may be due to more persistent patterns of food handling or acquisition among these populations. There is a need to better understand where in the farm-to-fork continuum minority populations are experiencing greater risks for exposure to these pathogens. 

## 3. Food Safety Knowledge and Practices among Minority and Low Socioeconomic (SES) Populations

Some of what we know about safe food handling knowledge and behaviors among minorities and low SES populations derives from consumer food handling surveys conducted among the general public with minority populations represented at what may or may not be equivalent to their representation in the general population. Additionally, if surveys were offered in English only there is a possibility that some minority populations were underrepresented. When the US Food Safety Survey began inclusion of a Spanish version, Hispanic participation increased from 5.4% to 15.3% over a ten year period although it subsequently dropped to only 11.8% five years later [59]. Therefore while such surveys may give us some insight into minority and low income knowledge and behavior in comparison to other segments of the population, these limitations must be considered in drawing conclusions from them. A meta-analysis of consumer surveys found that consumers with lower incomes and those with less than a high school education had good hygiene practices that exceeded their knowledge of safe food handling. That is to say, while they may not have reported knowing why they would perform certain practices, they did in fact perform those practices. It also found that high income individuals were more likely to consume raw foods (including raw beef), generally had less knowledge of hygiene than other demographics and were more likely to engage in practices that resulted in cross-contamination [54]. Similarly, it has been found that those with a graduate education and those with income greater than $75,000 were most likely to eat pink ground beef and only 10.4% of African American consumers reported eating pink ground beef, compared to 18.5% of Caucasians [53]. These findings are consistent with data that indicate that Caucasians are more likely than consumers of other race/ethnicities to suffer from *E. coli* O157:H7 infections [10,13]. Alternatively, an examination of safe food handling and consumption trends over a 22 year period found that African American and Hispanic consumers had higher risky food consumption than Caucasians, while Hispanics demonstrated significantly lower cross contamination than consumers of all other demographics [59]. Finally, while not traditionally considered a “food safety practice”, a recent survey of consumers in FoodNet sites was conducted to determine exposure of children to raw meat and poultry in the grocery store and shopping cart. Exposure of children to raw meat and poultry was higher for Hispanic and African American children when compared to Caucasian children. Lower household income and lower level of parental education were also associated with greater exposure of children to raw products [60].

Multiple researchers have targeted low income and minority populations to better understand their safe food handling knowledge and behaviors using focus groups [19,22], observational [61] and survey research designs [20,21,23]. Surveys of food safety knowledge and behaviors of consumers eligible for federal food subsidy benefits have identified both knowledge and safe behaviors lacking in this population [20,21,23]. Many respondents incorrectly reported believing that freezing would kill bacteria that caused foodborne illness and that food that can make you sick will always smell/taste bad. Additionally, incorrect knowledge and behavior regarding proper cooling and holding of food was reported by multiple researchers [21,23]. A nationwide survey of almost 1600 Women, Infant and Children (WIC) clients found Caucasian respondents had greater food safety knowledge than Hispanic respondents, consistent with earlier findings in a more localized survey with respondents in Arizona [20,21]. The same nationwide study also found that African American respondents had lower food safety behavior scores than those in any other racial or ethnic group and that thermometer usage was extremely uncommon (7.7%) among the entire population surveyed [20]. Focus group research with similar populations confirmed resistance of this population to use of thermometers [19,22]. The exploratory nature of focus groups also allowed researchers to identify unique barriers to safe food handling among these populations including lack of dishwashers and cutting boards [22] as well as extended travel time to obtain groceries [19]. The use of focus groups further identified unique practices reported among minority ethic/racial consumers including cooking turkey overnight as well as washing raw poultry either in water or acid/water rinses (*i.e.*, vinegar or lemon juice) [19]. It would be valuable to determine whether the challenges and behaviors found in focus group studies are commonly reported by larger samples of these populations.

A number of studies focused on the knowledge and behaviors of low income Puerto Rican consumers in the Northeast U.S. [17,18,61,62]. It was generally found that this population reported good knowledge of the need to wash hands properly and wash cutting boards, but they did not report utilizing thermometers to determine doneness of meat. The ability to speak English, a higher level of education and employment were all associated with increased familiarity with the terms cross-contamination and pasteurization [17]. It was further found, however that reported knowledge did not match actual handling behaviors, particularly with respect to use of proper thawing and appropriate hand washing [18]. Interestingly the observational research identified that with family income <$1,000 per month participants were less likely to use cutting boards, consistent with previous research that had identified lack of cutting boards as a potential barrier to safe food handling [22]. The researchers also observed kitchens to commonly be crowded with items unrelated to food preparation and lack of paper towels and soap [18].

Overall it would appear that research which focuses on the knowledge and behaviors of vulnerable populations (as opposed to the general population) provides much richer data regarding the food safety knowledge as well as potential barriers to safe food handling among these populations. There is a need to determine the prevalence of findings from smaller qualitative research among larger populations of minority and low SES consumers. There is also a need to better understand whether some barriers to safe food handling are cultural or simply due to lack of resources among low SES populations. If unsafe food handling practices such as lack of cutting boards, thermometers, soap and paper towels or improper hand washing are because of cultural beliefs or traditions there is a need to educate these populations with culturally appropriate materials about these importance of these practices. If however, it is a lack of resources due to poverty that cause some consumers to prepare food without basic necessities for proper sanitation there may be a need for a more direct public health intervention to increase the availability of these resources to low SES populations. 

## 4. Retail Food Safety for Minority and Low Socioeconomic Populations

In 2009 the U.S. Department of Agriculture released a report to congress which defined a food desert as a U.S. census tract which met both low-income and low-access criteria [27]. Low access was defined as greater than one mile (1.6 km) away from a supermarket in urban areas, assuming walkability, or greater than 20 miles (32.2 km) away in a rural area. Supermarkets and large grocery stores which provided high access were defined as food stores with at least $2 million in sales that contained all the major food departments found in a traditional supermarket. Therefore, while low access areas may have access to retail food outlets, these retail food outlets are likely small independently owned corner markets and convenience stores. This is consistent with a large body of research in public health nutrition [24,25,63,64,65]. A 2012 USDA report found that areas with high levels of poverty are more likely to be food deserts and with the exception of very dense urban areas, the higher the percentage of minority population, the more likely the area is to be a food desert [28]. 

From the perspective of food safety, if we assume that food access by low socioeconomic and minority populations is more likely to be through small, independent retailers than large chain supermarkets, the question arises whether small independent retailers pose a greater risk to food safety and sanitation than chain supermarkets. 

### 4.1. Microbial Quality of Food Available at Retail in Food Deserts

There is a paucity of data regarding the microbial quality and/or safety of foods available in small independently owned markets located in food deserts. One longitudinal study was performed over a fifteen month period comparing product quality in three supermarkets in high socioeconomic status (SES) census tracts and one supermarket and two independently owned grocery stores in low SES census tracts. Higher microbial loads were found on produce from markets in low SES areas. Specifically, ready to eat (RTE) bagged greens, strawberries and cucumbers had significantly higher yeast and mold counts (Y&M) and RTE greens and strawberries also had significantly higher Aerobic Plate Counts (APC) [30]. A larger cross-sectional study over a two year period sampled a range of perishable food products from retail food stores present in census tracts representing each of the following demographic categories: (1) Caucasian, (2) African American, (3) Asian, (4) Hispanic, (5) high SES and (6) low SES. Results indicated increased risks for improperly held eggs in markets in Asian census tracts as well as increased risks for fecal coliform contamination on RTE greens, herbs and fruit purchased in markets in Asian or low SES census tracts. Sandwiches from markets in Asian tracts were also significantly more likely to be contaminated with fecal coliforms when compared to sandwiches from markets in Caucasian census tracts. Additionally, while temperatures at time of sampling were not significantly different, APCs were significantly higher in milk samples from low SES and Hispanic tracts when compared to milk samples from high SES tracts [29]. Limitations of these types of studies include the large number of samples which must be tested in order to obtain statistically significant results. Given the sporadic nature of the contamination of food with pathogens such as *E. coli* O157:H7 and *Listeria*, sampling studies at the retail level would need to be extremely large to detect differences in risk exposure for these pathogens. Retrospective studies of where food was purchased by those who did become ill from such pathogens are needed to determine if the food desert presents a greater risk of exposure. One such study that linked increased listeriosis with increased social deprivation also found that when compared to the general public, those with listeriosis were less likely to purchase foods from supermarkets and more likely to purchase food from convenience and smaller local stores [6].

In addition to poorer quality mainstream food products, a potential risk for populations accessing food from small, particularly ethnic, retailers may be the presence of unique cultural foods, about which safety of the products is unknown. There is precedence for this with the example of fresh Mexican cheese consumed by Hispanic populations resulting in increased listeriosis among those populations [15,46,48]. A survey of Public Health Inspectors found that 60% of respondents reported at least one such specialty food product that they did not feel confident about their knowledge of its safety and 64% of respondents could identify at least one such specialty product about which they did not feel there is enough food safety information currently available [66]. 

### 4.2. Qualitative Research with Consumers, Small Food Retailers and Food Safety Professionals

An alternative way to determine levels of sanitation and safety in small markets is through qualitative research with low income consumers, store owners and inspectors. In one such study, consumers reported store cleanliness to be associated with the perception of fresh food and larger chain stores were perceived as cleaner than smaller, non-chain stores [67]. A survey of owners and managers of non-supermarket food retailers found that a percentage of retailers reported “self-supplying”—that is purchasing product from a supermarket or warehouse and transporting it themselves to their store. Milk was reported as self-supplied by 15% of the retailers interviewed and fresh fruits and vegetables were supported as self-supplied by 78% of the retailers [68]. This transport of food in unrefrigerated, personal vehicles between retail outlets certainly represents an unsafe branch in the farm to fork continuum that consumers who purchase these products are exposed to. A survey regarding ethnic food safety found that with respect to ethnic retail food facilities food safety professionals had concerns similar to those in mainstream retail facilities, however in addition to those concerns the food safety professionals also cited vermin as a concern more than 10% of the time for ethnic food retail facilities [69]. 

### 4.3. Food Safety Code Compliance by Retail Facilities in Food Deserts

Food retailers that operate in underserved areas with high poverty levels face a number of barriers and challenges. These have been identified as including, but not limited to, high costs associated with security and insurance, challenges to recruitment and retention of employees as well as transportation infrastructure [70]. In urban areas congestion and very small streets may present challenges to delivery, especially of perishable products, while in rural areas long distances from distribution centers may make delivery cost prohibitive. Additionally, it is known that food desert census tracts tend to have smaller populations with lower incomes (and therefore potentially an inadequate demographic base to support a medium or large sized market) as well as higher rates of abandoned or vacant homes [70]. Abandoned or vacant homes may lead to sanitation challenges in that they may serve as a breeding ground for pests and/or rodents. It has been suggested that poor infrastructure, crime and employee turnover likely all contribute to challenges for small retailers [71]. In addition to time and money, Yapp *et al*. [72] identified potential barriers to food safety compliance to include lack of trust in food safety regulations and compliance, as well as lack of motivation, knowledge and understanding of food safety legislation. These findings would all imply the likelihood that small independent markets would have more critical and non-critical code violations. Darcey and Quinlan [32] used GIS technology to map publically available critical health code violations (CHV) in retail facilities across a range of population demographics in Philadelphia, Pennsylvania. Overall, it was found that food service facilities in higher poverty areas had a greater number of facilities with at least one CHV and had more frequent inspections than facilities in areas with lower poverty. Additionally, CHV rates in census tracts with high Hispanic populations were greater than for CHV rates in tracts of any other population demographic. However, it was also seen that facilities in lower poverty areas had the highest average number of CHV per inspection, but a greater number of days between inspections, which is counterintuitive to what would be expected if facilities in low poverty areas had more CHVs [32]. These results indicate that while GIS technology may have potential applications to exploring relative safety and sanitation of retail facilities, the technology is dependent on health inspection data to be completely objective and not influenced by potential inspector bias [73]. The limitation to the use of this technology is the assumption that the number of critical code violations and/or facility overall “scores” are true predictors of food safety. The benefit of this technology is that it is less labor intensive than microbiological testing and much of the data may already be available through inspection records. 

Overall, research utilizing very different methods (microbial sampling, qualitative research, comparison of critical code violations) indicates sanitation and refrigeration challenges for small retailers operating in low socioeconomic areas. The limited amount of data however, makes it impossible to draw conclusions as to whether or not retail food access may be contributing to higher rates of foodborne illnesses among populations who access their food from these types of retailers. More retrospective studies such as the one by Gillespie *et al*. [6] may provide more insight as to whether or not the food desert environment is contributing to greater rates of foodborne illness. 

Attempts to combat high rates of obesity and chronic diseases associated with it have included many initiatives to increase retail access of affordable, nutritious food in food deserts [74,75,76]. Seemingly left out of the conversation is the fact that many of the nutritious, healthy foods being promoted are perishable products. This is especially of concern in light of increased outbreaks and incidences of foodborne illness due to fresh produce [77]. Efforts to increase the presence of perishable products in stores, neighborhoods and homes that may lack the infrastructure to ensure proper refrigeration, sanitation and pest control may result in unintended consequences in the form of foodborne illness or at the very least, wasted perishable products. 

## 5. Food Service and Minority and Low Socioeconomic Populations

Research has found that residents of low socioeconomic status (SES) areas and particularly areas with higher percent African American in the population have greater access to smaller, independently operated food markets and fast-food/take-out restaurants compared to those of high SES [24,25,63,78]. This differential access may be an increased food safety risk for low income and minority populations for two reasons. The first is that surveillance of foodborne disease outbreaks has found that 68% of outbreaks associated with a single place of food preparation were associated with a restaurant or deli while only 9% were associated with food prepared in a private home. Similarly, 63% of norovirus outbreaks (the most prevalent cause of outbreaks with a single confirmed or suspected etiology) were found to be associated with a restaurant or deli [79]. Because norovirus is not routinely identified in clinical laboratories and therefore not monitored by FoodNet, there is no data available regarding incidence rates of norovirus infection among minority and low income populations and the general public. If, as research suggest, low socioeconomic and minority populations disproportionately rely on fast food and take-out restaurants as a regular part of their diet, they may have greater exposure to food safety risks associated with food service facilities, including norovirus as well as other viral and bacterial pathogens commonly associated with outbreaks.

A second reason that increased access to fast food/take out restaurants by low SES and minority populations may put these populations at greater risk for foodborne illness is if they disproportionately rely on independent ethnic restaurants. An examination of foodborne illness outbreak data from 1990 to 2000 and found that outbreaks associated with ethnic foods rose from 3% to 11% and the highest number of outbreaks associated with ethnic foods (43%) occurred in restaurants [80]. Online health inspection reports for 500 independent restaurants in a selected area of Kansas indicated that ethnic restaurants had significantly more critical and non-critical violations than non-ethnic restaurants [31]. Specifically, increased violations were reported in categories including time and temperature controls, physical facility maintenance, protection from contamination and demonstrated knowledge. A comparison of critical and non-critical code violations between independent ethnic, chain ethnic, independent non-ethnic and chain non-ethnic facilities in Kansas found that independent ethnic restaurants had significantly greater numbers of both critical and non-critical violations and increased number of inspections when compared to all other groups [33]. Similarly, in the U.K. an examination of forty ethnic retail food businesses found that many had problems meeting minimum food hygiene and safety standards (*i.e.*, dirty: floors (48%), work surfaces (38%), equipment (65%) and lack of disinfectants (58%)). Additionally, it was found that only 53% of owners/managers reported to fully understanding inspectors’ reports and their expectations [81]. Other studies have identified the lack of inspectors’ knowledge about the safety of particular ethnic food products as well as a lack of food safety education material utilizing ethnic cuisine as areas which need to be addressed to increase the safety of ethnic food [69,82]. Potential food safety risks among ethnic food service facilities are certainly not limited to low income or minority populations, as ethnic food products have demonstrated a great appeal to a wide range of consumers. They are addressed here with emphasis on independent ethnic facilities because of increasing public health research that indicates that residents of food desert environments may rely on them more than the general public [26].

There has been no research to date which has directly examined the safety of food service facilities in the food desert environment as compared to food service facilities available to populations of higher income. Given the high rate of foodborne outbreaks associated with foodservice, increased dependence of populations living in food deserts on foodservice, and evidence that both independent ethnic restaurants [31,33] and retail food facilities in the food desert environment [29,30] may face greater challenges to food safety and sanitation, it seems that this is an area which needs further exploration to determine if retail foodservice facilities are contributing to increased rates of some foodborne illnesses by minority and low SES populations.

## 6. Conclusions

While much more is known today than even a decade ago about foodborne illness and food safety risks for low SES and minority populations in the U.S. and Europe, there remain many questions and areas where there is need for further research. With respect to incidence rates of foodborne illness there remains a need to better identify whether persistently higher levels of foodborne infections caused by *Salmonella*, *Shigella* and *Campylobacter* are a result of underlying cultural food handling practices or instead due to socioeconomic factors related to general poor sanitation, pest infestation and challenges to proper refrigeration both at the level of the consumer and retail access. *Campylobacter* presents a unique challenge and there is a need to better understand why rates appear to be consistently lower among African Americans but higher among Hispanics and Asians when compared to Caucasians. The possibility of immunity due to repeated exposure of this population to low levels of campylobacters or due to high levels of exposure in early childhood needs further exploration. There is also a need to consider sources other than food as potential routes of infection in light of recent research [42,43,44]. 

With respect to food handling among minority and low SES populations there is a need to continue to identify unique barriers to safe food handling as well as to determine how prevalent such barriers are in larger minority and low SES populations. If scarcity of resources (*i.e.*, cutting boards, paper towels, disinfectants, soap, and thermometers) is widespread and is a function of low SES then this needs to be identified and acknowledged through either education and/or public health interventions. There is also a need to address food safety knowledge gaps which have already been identified among these populations using culturally appropriate food safety education tools. 

With respect to retail food access, policy changes intended to increase consumption of healthy perishable products in food desert areas need to also ensure proper refrigeration, transportation and storage in small independent markets. Since small markets do not possess the knowledge or experience of food microbiologists there is a need to include the food safety and food processing communities in efforts to increase the nutritional quality of food available to low SES and minority racial and ethnic populations. There is a need for increased food safety education efforts to reach small business owners who operate either foodservice or food retail facilities in the food desert environment. Such education will need to address language, cultural and infrastructure limitations that these independent retailers may experience.

Finally with respect to the high access to, and reliance on, fast food and takeout food service by low income minority populations there is a need to better understand food safety risks associated with food service facilities located in the food desert environment. 
